# Leadless pacemakers at 5-year follow-up: the Micra transcatheter pacing system post-approval registry

**DOI:** 10.1093/eurheartj/ehae101

**Published:** 2024-03-01

**Authors:** Mikhael F El-Chami, Christophe Garweg, Nicolas Clementy, Faisal Al-Samadi, Saverio Iacopino, Jose Luis Martinez-Sande, Paul R Roberts, Claudio Tondo, Jens Brock Johansen, Xavier Vinolas-Prat, Yong-Mei Cha, Eric Grubman, Pierre Bordachar, Kurt Stromberg, Dedra H Fagan, Jonathan P Piccini

**Affiliations:** Division of Cardiology, Section of Electrophysiology, Emory University, Atlanta, Georgia; Department of Cardiovascular Sciences, Universitaire Ziekenhuizen Leuven, Leuven, Belgium; Department of Cardiologic Medicine, Centre Hospitalier Regional Universitaire de Tours—Hopital Trousseau, Tours, France; Department of Medicine, King Salman Heart Center—King Fahad Medical City, Riyadh, Saudi Arabia; Department of Clinical Electrophysiology & Cardiac Pacing, Centro Cardiologico Monzino, IRCCS, Department of Biomedical, Surgical, and Dental Sciences, University of Milan, Milan, Italy; Arrhythmia Unit, Cardiology Service, University Clinical Hospital of Santiago de Compostela, CIBER-CV, IDIS, Santiago de Compostela, Spain; Department of Medicine, University Hospital Southampton NHS Foundation Trust, Southampton, United Kingdom; Monzino Cardiac Center, IRCCS, Department of Clinical Sciences and Community, University of Milan, Milan, Italy; Department of Cardiology, Odense University Hospital, Odense, Denmark; Arrhythmia Unit, Hospital de la Santa Creu I Sant Pau, Barcelona, Spain; Department of Cardiovascular Medicine, Mayo Clinic, Rochester, MN, USA; Section of Cardiovascular Medicine, Yale University School of Medicine, New Haven, CT, USA; Cardio-Thoracic Unit, Bordeaux University Hospital, Pessac-Bordeaux, France; Medtronic, Inc., Mounds View, MN, USA; Medtronic, Inc., Mounds View, MN, USA; Electrophysiology Section, Duke Clinical Research Institute, Duke University Medical Center, Durham, NC, USA

**Keywords:** Leadless pacing, Long-term outcomes, Pacemaker, Bradycardia, Clinical trial

## Abstract

**Background and Aims:**

Prior reports have demonstrated a favourable safety and efficacy profile of the Micra leadless pacemaker over mid-term follow-up; however, long-term outcomes in real-world clinical practice remain unknown. Updated performance of the Micra VR leadless pacemaker through five years from the worldwide post-approval registry (PAR) was assessed.

**Methods:**

All Micra PAR patients undergoing implant attempts were included. Endpoints included system- or procedure-related major complications and system revision rate for any cause through 60 months post-implant. Rates were compared through 36 months post-implant to a reference dataset of 2667 transvenous pacemaker patients using Fine–Gray competing risk models.

**Results:**

1809 patients were enrolled between July 2015 and March 2018 and underwent implant attempts from 179 centres in 23 countries with a median follow-up period of 51.1 months (IQR: 21.6–64.2). The major complication rate at 60 months was 4.5% [95% confidence interval (CI): 3.6%–5.5%] and was 4.1% at 36 months, which was significantly lower than the 8.5% rate observed for transvenous systems (HR: .47, 95% CI: .36–.61; *P* < .001). The all-cause system revision rate at 60 months was 4.9% (95% CI: 3.9%–6.1%). System revisions among Micra patients were mostly for device upgrades (41.2%) or elevated thresholds (30.6%). There were no Micra removals due to infection noted over the duration of follow-up. At 36 months, the system revision rate was significantly lower with Micra vs. transvenous systems (3.2% vs. 6.6%, *P* < .001).

**Conclusions:**

Long-term outcomes with the Micra leadless pacemaker continue to demonstrate low rates of major complications and system revisions and an extremely low incidence of infection.

## Structured Graphical Abstract

**Structured Graphical Abstract:**
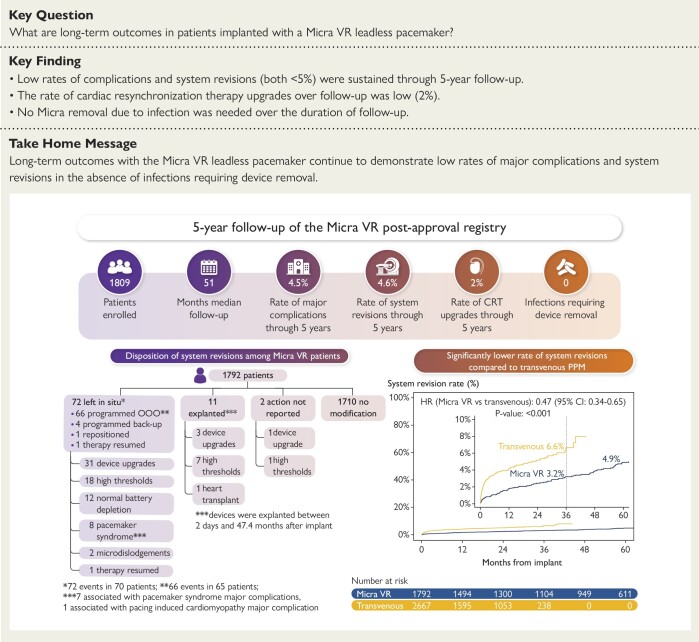
Five-year outcomes of patients implanted with a Micra VR leadless pacemaker. The flowchart on the left depicts the disposition of system revisions. The figure on the right shows a comparison of system revision rates for Micra VR and a historical transvenous pacemaker cohort. CI, confidence interval; CRT, cardiac resynchronization therapy; HR, hazard ratio.

**See the editorial comment for this article ‘Leadless and scarless pacing: towards symbiotic nanogenerators’, by P.E. Vardas, https://doi.org/10.1093/eurheartj/ehae124.**

## Introduction

Leadless pacemakers (LP) are an established pacing alternative to traditional transvenous permanent pacemakers (TV-PPM) for the treatment of brady-arrhythmias.^1^ Early data from the Micra transcatheter pacing system (TPS) investigational device exemption (IDE) study showed a high implant success rate (>99%) and low complication rate when compared to a historical cohort of TV-PPM patients.^2^ Data from the Micra post-approval registry (PAR) demonstrated consistent outcomes and in addition, showed a lower rate of complications mainly driven by reductions in the rate of Micra implant perforations.^3,4^ One-year follow-up of patients enrolled in the IDE study and PAR showed a sustained benefit in terms of a reduction in complications with Micra when compared to TV-PPM.^3,5^ Furthermore, the Micra coverage with evidence development study (CED), a Medicare mandated study where outcomes are based on claims data, showed that the benefits of LP are accentuated during intermediate-term follow-up (2 and 3 years).^6,7^ Specifically, LPs were associated with a 32% reduction in total complications mainly driven by a 41% lower reintervention rate. As with any new technology, long-term monitoring of the performance of this new and innovative technology is paramount. The Micra PAR was designed to prospectively follow enrolled patients for 9 years with two main purposes: (i) to determine the reliability and safety of these devices during long-term follow-up, and (ii) to report on the management of the device life cycle from implant to end of life (EOL). In this manuscript, we report on the updated and long-term performance of the Micra VR LP in a worldwide real-world setting through 5-year follow-up.

## Methods

### Study design

The design of the Micra ventricle, rate modulation/sensor (VR) PAR has been described previously.^4^ In brief, the Micra VR PAR is a non-randomized, prospective registry study designed to assess the performance of the Micra VR system when used in real-world clinical practice throughout the device’s life cycle. The study enrolled patients with class I or II indications for pacing^8,9^ with no co-morbidity restrictions and will follow patients up to 9 years post-implant. Ethics committee approval was obtained at each participating centre as applicable. A Clinical Events Committee (CEC) of independent physicians adjudicated all system- and procedure-related adverse events.

### Patients and procedures

All patients who were intended to be implanted with the Micra VR device at each study centre were eligible. Patients were considered enrolled upon providing written informed consent. Patients enrolled in a pre-market trial could also enrol in the Micra VR PAR (ClinicalTrials.gov identifier: NCT02536118) for long-term follow-up but were excluded from the present analysis.

Following enrolment, patients underwent Micra VR (model MC1VR01, Medtronic, Inc. Minneapolis, MN, USA) implant attempt using standard practice and are followed according to their centre’s routine care practices. Patient and device status is assessed at implant, pre-hospital discharge, 30 days, and at least annually thereafter. All system- and procedure-related adverse events, all system revisions (e.g. device replacements), and all patient deaths regardless of cause are to be reported immediately following centre awareness. Study enrolments occurred between July 2015 and March 2018. The 9-year follow-up period is currently ongoing. Earlier term outcomes through 12 months post-implant were reported previously.^3^

### Endpoints

The objective of this analysis was to assess system- or procedure-related major complications, system revision for any reason, and all-cause mortality through 5 years (60 months). Major complications were defined as system- or procedure-related adverse events that resulted in death, permanent loss of device function, hospitalization, prolonged hospitalization by ≥48 h, or system revision. The CEC reviewed and adjudicated all system- and procedure-related events to determine relatedness and whether any related events met any of the major complication criteria. System revisions included any invasive modification of the device (e.g. replacement, revision, and explant) or date the device was programmed off [NO pacing, sensing or rate response (OOO) mode]. Electrical performance at implant and 12-month follow-up intervals was also characterized.

For comparative purposes, major complications and system revisions were compared to a data set of 2667 patients with *de novo* pacemakers from 6 Medtronic sponsored studies of dual-chamber pacemakers (historical TV-PPM cohort) that included five pre-market and one post-market study completed between 2000 and 2012.^10^ Each study had an independent adverse event adjudication committee and, as described previously,^2^ events related only to the right atrial lead were excluded to approximate a single-chamber data set.

### Statistical methods

The study database was frozen for analysis on 16 April 2023 and included all study visits and reported events as of 1 March 2023. Eight patients included in the previously reported cohort^3^ were excluded due to inadequate informed consent identified during sponsor monitoring visits. Summary statistics were obtained and reported using mean ± standard deviation (SD) or median and interquartile range (IQR) for continuous variables and frequencies and percentages for categorical variables. *T*-tests (continuous variables) or Fisher’s exact test (categorical variables) were used to compare baseline and medical history variables between Micra VR PAR and historical TV-PPM cohort. System- or procedure-related major complication rates were computed overall and by category using frequencies and percentages (within 30 days of implant) or cumulative incidence functions (CIFs) under the competing risk of death unrelated to the system or procedure at 12, 36, and 60 months post-implant. CIFs were also used to compute system revision rates through 60 months. Similarly, CIFs were used to compute device out-of-service rates through 60 months for elevated pacing threshold or battery depletion and other reasons (*e.g.* system upgrade) under the competing risk of death. The Fine–Gray competing risk model was used to compare the risk for system- or procedure-related major complications and system revisions for any reason between the 2667 in the transvenous group and patients in the Micra VR PAR with an implant attempt through 36 months implant (follow-up duration of the historical TV-PPM cohort). Day zero was defined as the day of implant attempt for time-to-event analyses.

To account for differences in baseline and co-morbidities between Micra VR PAR patients and the 2667 patients in historical TV-PPM cohort, propensity score overlap weights were used to derive adjusted hazard ratios (HRs) for the comparison of major complications and system revisions between Micra VR patients and transvenous patients. To compute the propensity scores, a logistic regression model was used to model the likelihood of receiving Micra VR given the variables displayed in Supplementary data online, *Table S1*. The resulting propensity scores were used to derive the overlap weight for each patient which could be used in weighted Fine–Gray models. Propensity score overlap weights place the most emphasis on patients considered most comparable and the least emphasis on patients least likely to be treated with the opposing therapy.^6^ Due to the presence of missing baseline and co-morbidity data required to derive the propensity scores, adjusted HRs were computed across 100 imputed datasets using the fully conditional specification approach^11^ and combined into a single estimate and 95% confidence interval (CI) using Rubin’s rule.^12^ The Supplement provides additional details on the propensity score analysis.

Electrical parameters were summarized at implant and 12-month intervals using means and SDs. Ventricular pacing percentage at last device interrogation was summarized using the median and IQR. Remaining battery longevity, standardized to 5 years post-implant, was projected using Monte Carlo methods by combining bench measured static current drain distributions combined with actual use conditions obtained from each patient’s last available device interrogation provided it was at least 183 days post-implant. The battery longevity model also assumed each patient would have 6, 30-min telemetry session per year. All analyses were performed with SAS version 9.4 (SAS Institute Inc., Cary, NC, USA) or the R statistical package (www.r-project.org).

## Results

### Patients and follow-up

A total of 1809 enrolled patients underwent implant attempts from 179 centres in 23 countries between July 2015 and March 2018 with a median follow-up period of 51.1 months (IQR: 21.6–64.2) and leading-edge follow-up of 86.7 months. Detailed baseline and implant characteristics have previously been described.^3^ Briefly, at implant, patients had a median age of 79 (IQR: 71–84) years, were 38.8% female, and had multiple co-morbidities (*Table 1*). The Micra VR device was implanted successfully in 1792 (99.1%) of the 1809 patients with the right ventricular septum being the most common implant location (65.1%).

**Table 1:** Baseline characteristics

Patient characteristics	Implant attempt (*N* = 1809)
**Age, years**	
Mean ± standard deviation	75.6 ± 13.4
Median (25th–75th percentile)	79.0 (71.0–84.0)
**Female sex**	38.8% (701/1808)
**Co-morbidities**	
Atrial tachyarrhythmias	75.9% (1373/1808)
CHF	15.4% (279/1808)
COPD	9.8% (177/1808)
CAD	22.0% (398/1808)
HTN	64.9% (1173/1808)
Diabetes	26.5% (479/1808)
Renal dysfunction	21.5% (389/1808)
Dialysis	7.9% (143/1808)
**No. of co-morbidities** ^a^	
None	6.7% (122/1808)
1	21.1% (382/1808)
2	28.4% (513/1808)
3 or more	43.8% (791/1808)
**Condition that precludes the use of TV-PPM**	24.0% (433/1807)
**Prior CIED**	15.2% (275/1808)
**Pacing indication (%)**	
Bradyarrhythmia with AF	62.6% (1128/1802)
Sinus node dysfunction	9.6% (173/1802)
AV block	11.7% (210/1802)
Syncope	13.5% (244/1802)
Other	2.6% (47/1802)
**Pericardial effusion risk level (%)** ^b^	
Low	71.0% (1255/1767)
Medium	17.1% (303/1767)
High	11.8% (209/1767)

^a^Co-morbidities include those listed under co-morbidity sub-category.

^b^Pericardial effusion risk level calculated per Piccini *et al*.^13^

AF, atrial fibrillation; AV, atrioventricular; CHF, congestive heart failure; CIED, cardiac implantable electronic device; COPD, chronic obstructive pulmonary disease; CAD, coronary artery disease; HTN, hypertension; TV-PPM, transvenous permanent pacemaker.

### Safety

A total of 85 major complications adjudicated as related to the Micra VR system or procedure were reported in 79 patients during the entire follow-up period with 83 in 77 patients occurring within 60 months of implant for a 5-year rate of 4.5% (95% CI: 3.6%–5.5%) (*Table 2*). Most major complications (58.8%, 50/85) occurred within 30 days of the implant procedure and largely included procedure-related events such as thrombosis, groin puncture events, and pericardial effusions/perforations in addition to pacing capture and elevated threshold events. Details regarding the pericardial effusion events have been reported previously.^3,13^ Longer-term major complications included pacing capture and elevated threshold events (14 following the acute 30-day period), pacemaker syndrome (seven following the acute 30-day period), and pacing-induced cardiomyopathy (five following the acute 30-day period). Of the pacemaker syndrome and pacing-induced cardiomyopathy events, 11 of 12 required a device upgrade. There were no telemetry issues and no premature battery failure issues reported.

**Table 2:** Major complications for patients with an attempted Micra VR implant procedure (n = 1809)

	Total events (total patients, cumulative %)				
Adverse event keyterm	30-days	12-months	36-months	60-months	All events
**Total events**	**50** (**45, 2.49%)**	**67** (**61, 3.43%)**	**78** (**72, 4.09%)**	**83** (**77, 4.47%)**	**85** (**79)**
**Thrombosis**	**2** (**2, .11%)**	**2** (**2, .11%)**	**2** (**2, .11%)**	**2** (**2, .11%)**	**2** (**2)**
Deep vein thrombosis	1 (1, .06%)	1 (1, .06%)	1 (1, .06%)	1 (1, .06%)	1 (1)
Pulmonary embolism	1 (1, .06%)	1 (1, .06%)	1 (1, .06%)	1 (1, .06%)	1 (1)
**Events at groin puncture site**	**10** (**10, .55%)**	**11** (**11, .61%)**	**12** (**12, .67%)**	**12** (**12, .67%)**	**12** (**12)**
Arteriovenous fistula	3 (3, .17%)	4 (4, .22%)	5 (5, .28%)	5 (5, .28%)	5 (5)
Incision site haemorrhage	2 (2, .11%)	2 (2, .11%)	2 (2, .11%)	2 (2, .11%)	2 (2)
Lymphatic fistula	1 (1, .06%)	1 (1, .06%)	1 (1, .06%)	1 (1, .06%)	1 (1)
Vascular pseudoaneurysm	2 (2, .11%)	2 (2, .11%)	2 (2, .11%)	2 (2, .11%)	2 (2)
Vessel puncture site haematoma	2 (2, .11%)	2 (2, .11%)	2 (2, .11%)	2 (2, .11%)	2 (2)
**Cardiac effusion/perforation**	**8** (**8, .44%)**	**8** (**8, .44%)**	**8** (**8, .44%)**	**8** (**8, .44%)**	**8** (**8)**
Cardiac perforation	1 (1, .06%)	1 (1, .06%)	1 (1, .06%)	1 (1, .06%)	1 (1)
Cardiac tamponade	6 (6, .33%)	6 (6, .33%)	6 (6, .33%)	6 (6, .33%)	6 (6)
Pericardial effusion	1 (1, .06%)	1 (1, .06%)	1 (1, .06%)	1 (1, .06%)	1 (1)
**Pacing issues**	**17** (**16, .88%)**	**23** (**22, 1.24%)**	**26** (**25, 1.42%)**	**29** (**28, 1.65%)**	**31** (**30)**
Device capturing issue/elevated thresholds	13 (13, .72%)	19 (19, 1.07%)	22 (22, 1.25%)	25 (25, 1.49%)	27 (27)
Device dislocation without embolization	2 (2, .11%)	2 (2, .11%)	2 (2, .11%)	2 (2, .11%)	2 (2)
Device embolization during implant	1 (1, .06%)	1 (1, .06%)	1 (1, .06%)	1 (1, .06%)	1 (1)
Undersensing	1 (1, .06%)	1 (1, .06%)	1 (1, .06%)	1 (1, .06%)	1 (1)
**Cardiac rhythm disorder**	**1** (**1, .06%)**	**2** (**2, .11%)**	**3** (**3, .17%)**	**3** (**3, .17%)**	**3** (**3)**
Cardiac arrest	1 (1, .06%)	1 (1, .06%)	1 (1, .06%)	1 (1, .06%)	1 (1)
Extrasystoles	0 (0, .0%)	1 (1, .06%)	1 (1, .06%)	1 (1, .06%)	1 (1)
Ventricular dyssynchrony	0 (0, .0%)	0 (0, .0%)	1 (1, .06%)	1 (1, .06%)	1 (1)
**Infection**	**4** (**4, .22%)**	**4** (**4, .23%)**	**5** (**5, .28%)**	**5** (**5, .28%)**	**5** (**5)**
Abdominal wall infection	1 (1, .06%)	1 (1, .06%)	1 (1, .06%)	1 (1, .06%)	1 (1)
Catheter site infection	1 (1, .06%)	1 (1, .06%)	1 (1, .06%)	1 (1, .06%)	1 (1)
Device-related infection	0 (0, .0%)	0 (0, .0%)	1 (1, .06%)	1 (1, .06%)	1 (1)
Haematoma infection	1 (1, .06%)	1 (1, .06%)	1 (1, .06%)	1 (1, .06%)	1 (1)
Sepsis	1 (1, .06%)	1 (1, .06%)	1 (1, .06%)	1 (1, .06%)	1 (1)
**Other**	**8** (**8, .44%)**	**17** (**17, .97%)**	**22** (**22, 1.27%)**	**24** (**24, 1.43%)**	**24** (**24)**
Acute pulmonary oedema	1 (1, .06%)	1 (1, .06%)	1 (1, .06%)	1 (1, .06%)	1 (1)
Blood pressure decreased	1 (1, .06%)	1 (1, .06%)	1 (1, .06%)	1 (1, .06%)	1 (1)
Cardiac failure	1 (1, .06%)	1 (1, .06%)	3 (3, .18%)	3 (3, .18%)	3 (3)
Cardiac failure congestive	0 (0, .0%)	1 (1, .06%)	1 (1, .06%)	1 (1, .06%)	1 (1)
Chest pain	1 (1, .06%)	1 (1, .06%)	1 (1, .06%)	1 (1, .06%)	1 (1)
Complications of device removal	1 (1, .06%)	1 (1, .06%)	1 (1, .06%)	1 (1, .06%)	1 (1)
Pacemaker syndrome	0 (0, .0%)	5 (5, .29%)	5 (5, .29%)	7 (7, .44%)	7 (7)
Pacing induced cardiomyopathy	0 (0, .0%)	2 (2, .12%)	5 (5, .30%)	5 (5, .30%)	5 (5)
Pulmonary oedema	1 (1, .06%)	1 (1, .06%)	1 (1, .06%)	1 (1, .06%)	1 (1)
Retroperitoneal haemorrhage	1 (1, .06%)	1 (1, .06%)	1 (1, .06%)	1 (1, .06%)	1 (1)
Syncope	1 (1, .06%)	1 (1, .06%)	1 (1, .06%)	1 (1, .06%)	1 (1)
Tricuspid valve incompetence	0 (0, .0%)	1 (1, .06%)	1 (1, .06%)	1 (1, .06%)	1 (1)

Notes: 1-month rate computed as patients with events divided by patients (1809). Longer-term rates are based on the cumulative incidence function.

### Infections

There were a total of nine all-cause infection events reported; five met the criteria for a major complication and the remaining four were observations (*Table 3*). Infection events occurred between 0 and 390 days post-implant. Of the five infection major complication events, three (sepsis, abdominal wall infection, and haematoma infection) were previously reported.^3^ The remaining two major complication infection events included a catheter site groin infection that resolved with antibiotics and a possible device-related infection. The possible device-related infection event occurred 390 days post-implant in a patient on haemodialysis. The patient underwent echocardiogram which found endocarditis and concern for pacemaker infection. The patient was given intravenous antibiotics and the infection resolved without further intervention. None of the infection events resulted in device removal.

**Table 3:** Infection events in Micra VR patients

Event no	Days post-implant	Event	Major complication	Outcome
1	0	Sepsis	Yes	Resolved, IV antibiotics
2	7	Haematoma infection	Yes	Resolved, IV antibiotics
3	7	Puncture site infection	No	Resolved, oral antibiotics
4	13	Groin infection	No	Resolved, oral antibiotics
5	17	Groin infection	No	Resolved, oral antibiotics
6	20	Catheter site infection	Yes	Resolved, IV antibiotics
7	25	Abdominal wall infection	Yes	Resolved, IV antibiotics
8	29	Postoperative wound infection	No	Resolved, no action taken
9	390	Device-related infection	Yes	Resolved, IV antibiotics

### All-cause mortality

There were 676 deaths reported for any reason during the follow-up period yielding a 5-year mortality rate of 39.5% (see Supplementary data online, *Figure S1*). Of the deaths, five were procedure related (of which two were due to cardiac perforation) which have been described in detail previously,^3^ 35 were classified as sudden cardiac, 113 were non-sudden cardiac, 345 were non-cardiac (including 15 from COVID-19), and 178 had an unknown classification (see Supplementary data online, *Table S2*).

### System revisions

**Figure 1:**
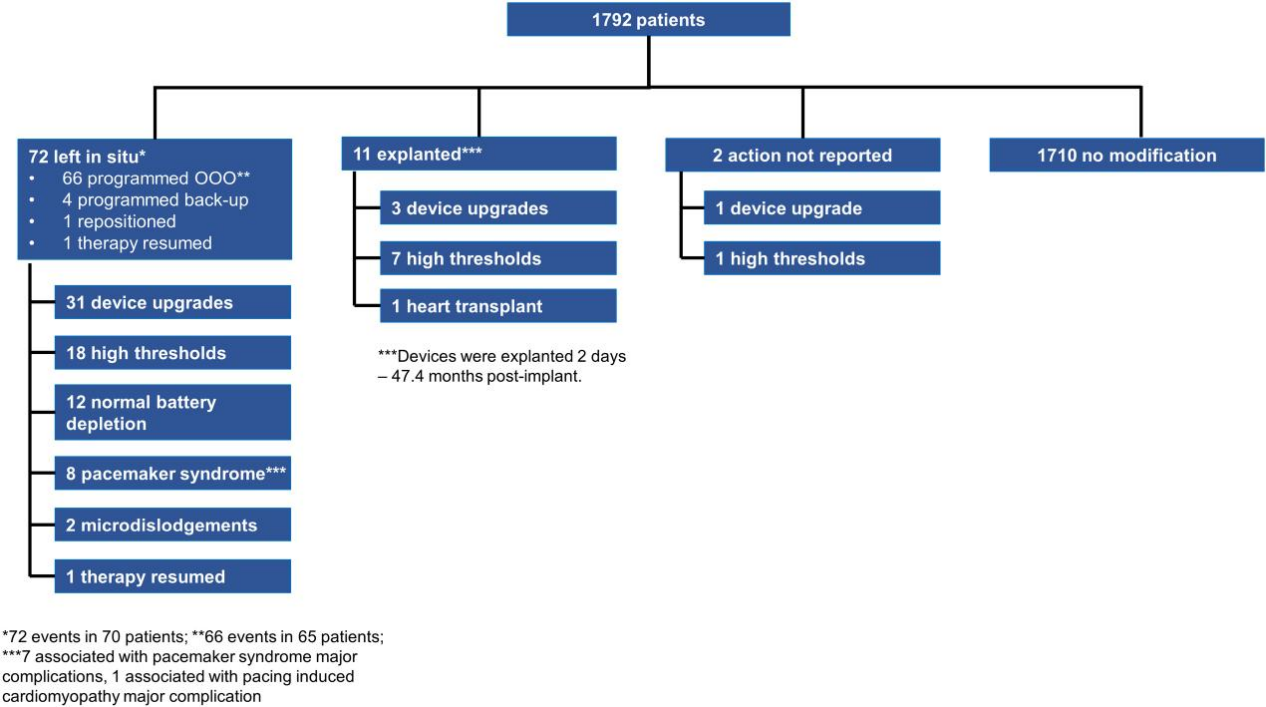
System revisions in Micra patients. Diagram depicting number of system revisions, action taken, and reason for revision

*Figure 1* provides a full accounting of the 85 Micra VR system revisions that were reported in 82 patients during the follow-up period. The all-cause system revision rate at 60 months was 4.9% (95% CI: 3.9%–6.1%). The three most common reasons for system revision included device upgrade (e.g. upgrade to dual-chamber transvenous pacing system or cardiac resynchronization therapy pacemaker [CRT-*P*]) in 35 patients, high pacing threshold (26 in 25 patients), and normal/expected battery depletion in the setting of baseline elevated thresholds (12 in 12 patients). The 12 system revisions for normal battery depletion occurred between 32.9 and 72.0 months post-implant in patients with a mean pacing capture threshold of 2.7 ± 1.0 V at .24 ms. There were eight system revisions for pacemaker syndrome which occurred between 2.3 and 56.0 months post-implant. Of these eight patients, five were upgraded to a CRT system, two received a transvenous dual-chamber pacemaker, and one received a Micra atrioventricular (AV) system. There was one system revision in which Micra therapy was resumed due to an infection of the patient’s transvenous device. On the day of Micra VR implant, the patient’s prior transvenous system was removed. The patient was upgraded to a CRT-P system 20.5 months post-implant and the Micra VR device was programmed to OOO mode. The patient then developed a cardiac implantable electronic device (CIED) infection 3.5 months later and their CRT-P system was removed and their Micra VR pacing therapy was enabled. The patient remains in follow-up.

The most common actions taken with the Micra VR system following system revision were to programme the device to OOO mode (66 cases in 65 patients) or explant the device [11 cases in 11 patients (including one heart transplant)]. Of the 11 Micra VR patients that had their Micra VR explanted, one occurred during a heart transplant, one was explanted surgically 42.1 months post-implant and the remaining nine were explanted percutaneously using either a Micra catheter or off-the-shelf snare between 2 days and 47.4 months post-implant. There was one major complication associated with explant [abdominal wall infection after the Micra device became entangled in an inferior vena cava filter (a contraindication for Mica implant) during explant, as previously described.^3^] There were four system revisions where the Micra VR system was programmed to backup pacing (e.g. VVI-40) following upgrade to a transvenous system.

There were 13 patients who had a second Micra device implanted within the right ventricle (RV) following system revision while abandoning the original Micra. There were no adverse events reported relating to device interaction during a median follow-up of 13.0 (IQR: 7.7–17.5) months following the implant of the second Micra device. However, one patient had their second Micra VR device programmed to OOO and was upgraded to a CRT-P due to a high threshold 2.4 months after their second Micra VR implant.

There were 33 patients upgraded to a CRT system between .4 months and 68.5 months post-implant. Of these, five upgrades occurred in patients who were implanted with a CRT system prior to receiving their Micra device. At 5 years, the rate of CRT upgrade was 2.0% (95% CI: 1.4%–2.8%) among all Micra patients and 1.7% (95% CI: 1.1%–2.5%) among the 1741 patients without a prior CRT system (see Supplementary data online, *Figure S2*).

Through 5 years post-implant, the cumulative rate of patients with their first device out of service for elevated pacing threshold or battery depletion was 2.1% with an additional 2.7% of devices out of service for other reasons (e.g. device upgrade) (see Supplementary data online, *Figure S3*). The remaining 95.2% of patients had their initial device still active at the time of death (37.5%) or at last follow-up (57.7%).

### Device electricals and pacing

**Figure 2:**
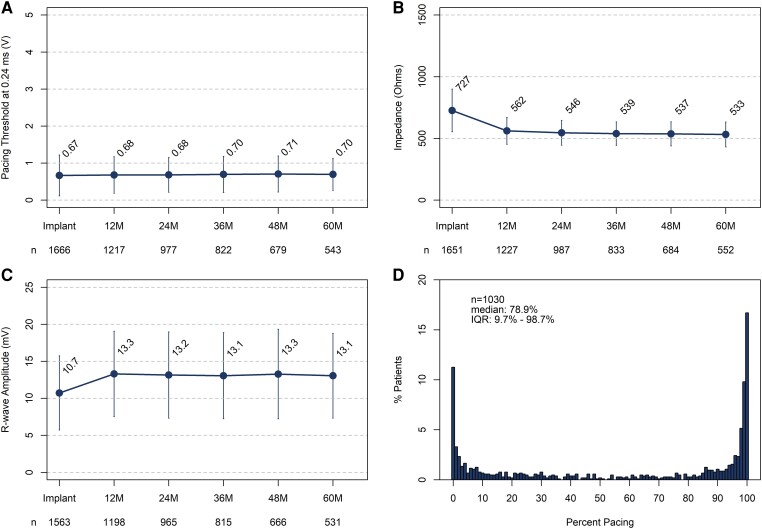
Summary of device electrical values and ventricular pacing. Average pacing capture threshold at .24 ms over time (*A*), average pacing impedance over time (*B*), average R-wave sensing amplitude over time (*C*), and distribution of ventricular pacing percentage at last device interrogation (*D*). Error bars represent ± standard deviation

The mean pacing capture threshold was .67 ± .55 V at .24 ms at implant and .70 ± .44 V at .24 ms at 60 months post-implant (*Figure 2A*). The mean pacing impedance was 727 ± 173 Ω at implant and 533 ± 101 Ω at 60 months (*Figure 2B*). Average sensing amplitude was 10.7 ± 5.0 mV at implant and 13.1 ± 5.7 mV at 60 months (*Figure 2C*). Median ventricular pacing percentage among the 1030 patients with device interrogation data available for analysis was 78.9% (IQR: 9.7%–98.7%) and was bimodally distributed (*Figure 2D*). Based on device use conditions from 920 patients, projected median remaining battery longevity after 5 years of follow-up was 6.8 years with 83.8% of patients having at least 5 years of additional projected battery life remaining.

### Comparison to historical control

**Figure 3:**
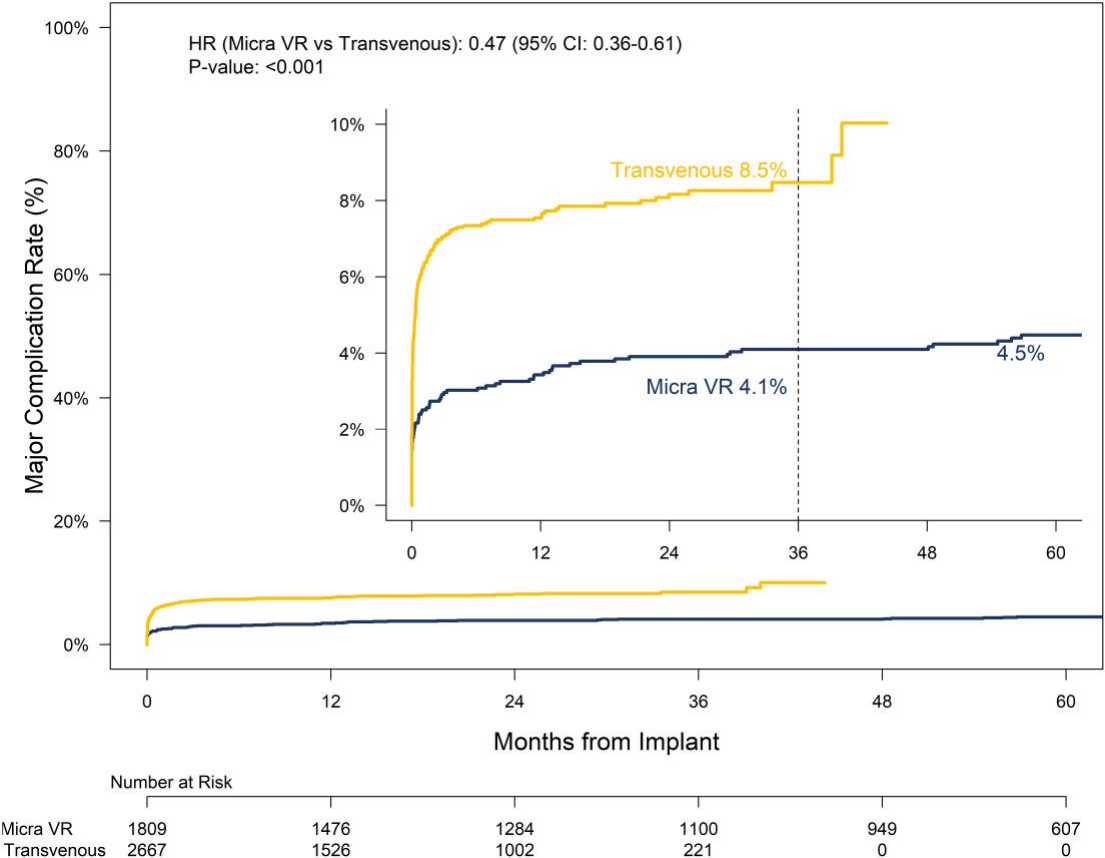
System or procedure-related major complication rates through 60 months post-implant for the Micra VR PAR and historical TV-PPM cohort. Sub-distributional hazard ratio based on data through 36-months post-implant as indicated by the dashed vertical line

**Figure 4:**
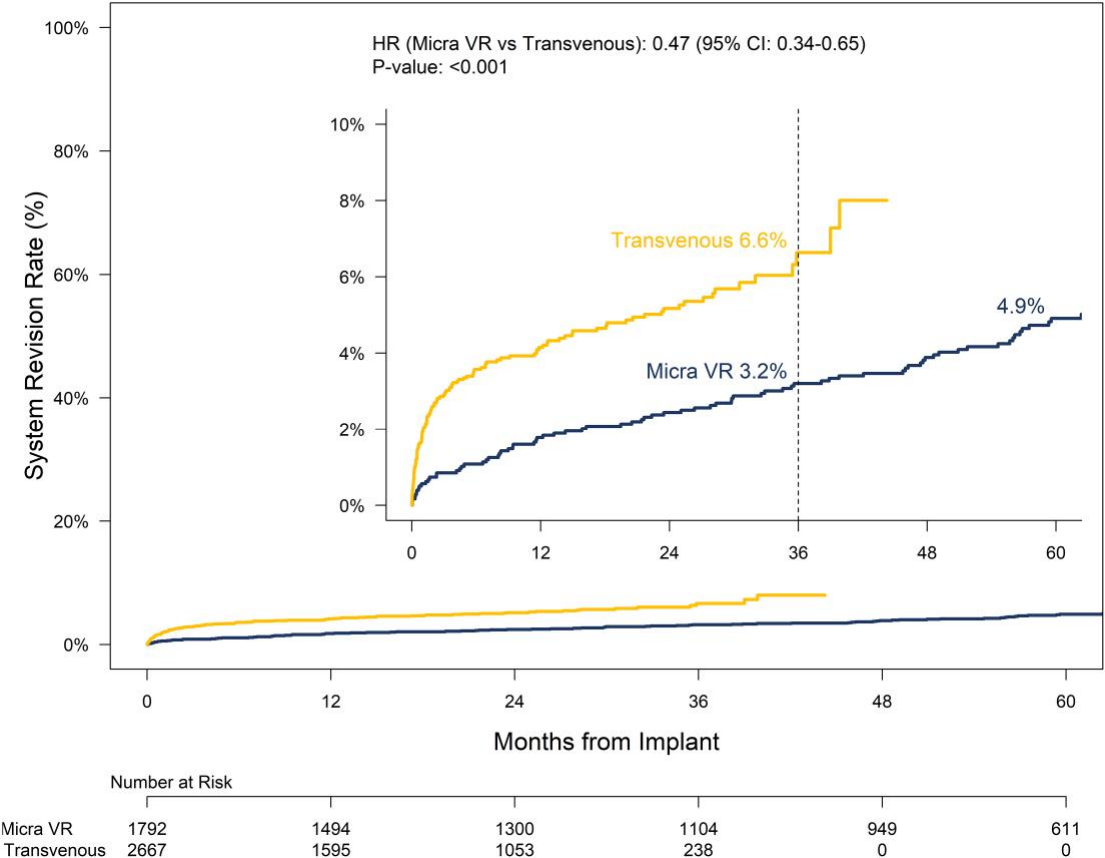
System revision rates for any cause through 60 months post-implant for the Micra VR PAR and historical TV-PPM cohort. Sub-distributional hazard ratio based on data through 36-months post-implant as indicated by the dashed vertical line. The impact of leadless pacing on the risk for system revisions was more pronounced within the first month following implant (HR: .28, 95% CI: .13–.61) compared to after 1 month (HR: .64, 95% CI: .44–.92)

Patients in the PAR tended to be older, were more likely to be male, and had a higher incidence of atrial fibrillation (AF), COPD, diabetes, and renal dysfunction than did the historical TV-PPM cohort (see Supplementary data online, *Table S1*). Through 36 months post-implant the major complication rate for Micra VR patients was 4.1% compared to 8.5% for the historical TV-PPM cohort (HR: .47, 95% CI: .36–.61, *P* < .001, *Figure 3*). The reduction in risk for major complications with Micra did not differ by timeframe (≤30 days vs. > 30 days; *P* = .43). Baseline characteristics were well balanced following propensity score weighting (see Supplementary data online, *Figure S4*). The reduction in the risk for major complication through 36 months was similar following propensity score adjustment (adjusted HR: .43, 95% CI: .29–.65, *P* < .001, Supplementary data online, *Figure S5*). There were 138 system revisions in 128 patients in the historical TV-PPM cohort (see Supplementary data online, *Table S3*). All-cause system revision rates through 36 months for Micra VR patients was 3.2% compared to 6.6% for the historical TV-PPM cohort (HR: .47, 95% CI: .34–.65, *P* < .001, *Figure 4*). The reduction in risk for system revisions with Micra was more pronounced within the first 30 days following implant as compared to after 30 days although the difference was not statistically different (HR: .28, 95% CI: .13–.61 vs. HR: .64, 95% CI: .44–.92, *P* = .061). Following propensity score adjustment, the risk for system revision remained lower for Micra VR patients compared to the historical TV-PPM cohort (adjusted HR: .48, 95% CI: .29–.78, *P* = .003, Supplementary data online, *Figure S6*).

## Discussion

There are several important findings from this global real-world analysis representing the longest longitudinal follow-up of LP to-date. First, the results of this study highlight the reliability of the Micra leadless pacemaker with low rates of complications and system revisions (both <5%) sustained through 5 years of follow-up. Second, the rate of CRT upgrade was low (2%) overall and was even less (1.7%) among patients without a prior CRT implant. Third, there were no devices removed due to infection. Finally, these findings shed light on Micra leadless pacemaker lifecycle management at the time of upgrade, revision, or battery depletion (Structured Graphical Abstract).

The observed 4.5% rate of major complications through 5 years provides reassurance on the long-term safety profile of the Micra leadless pacemaker. While early findings from both the IDE and PAR demonstrated favourable safety profiles that were sustained through 1 year,^3,5^ it is important when evaluating new devices to ensure that no new or unanticipated safety issues arise during extended follow-up. Most major complications occurred within 30 days, with minor increases beyond 12 months, including primarily elevated threshold events. Through 3 years, there was a 53% lower rate of observed major complications relative to TV-PPM, driven by absence of lead dislodgements and revisions. These findings align with recent results from the Micra CED study, which demonstrated sustained reductions in complications with Micra relative to TV-PPM through 3 years.^7^

The rate of system revision at 3 years was 3.2% which corresponds to a 53% lower rate when compared with TV-PPM (3.2% vs. 6.6%). This is comparable to the 41% lower rate of system revisions seen in the Micra CED study 3-year follow-up. The need to upgrade to CRT system was the most common reason for system modification, followed by elevated threshold and battery depletion (due to elevated thresholds). Overall, the need for CRT upgrade was low (2%) despite a median pacing burden of 78.9%. This finding may be surprising since the incidence of pacing induced cardiomyopathy (PICM) has previously been reported to range from 6% to 39%, depending upon the definition used to define PICM.^14,15^ It is possible that patient selection is the major reason for this low rate of CRT upgrade. Additionally, the preferential mid to high septal location of LP, which was the reported implant location in 65% of patients in this cohort, has been associated with a lower risk of PICM.^16^

A major finding of this study is the absence of device-related infection requiring LP removal. One of the major advantages of a LP is the absence of a subcutaneous pocket which eliminates/reduces the most common source of pacemaker infections. In addition, the small surface area and the potential for encapsulation likely contribute to the low predisposition to infection.^17^ In this long-term study, almost all infections observed were soft tissue infections that responded to antibiotics or conservative management. One dialysis patient had valve endocarditis with possible device involvement. The device was not removed and the patient recovered. The findings from the 5-year PAR data are consistent with early data supporting the concept that the risk of device infection is extremely low with LP. Furthermore, a previous analysis from this cohort suggested that Micra appears to be a safe pacing alternative for patients with CIED infection who undergo extraction, with no recurrent infections requiring device removal observed among the 105 patients studied.^18^

One of the major questions for LP is the management of these devices at the time of an upgrade or system modification and battery depletion. As expected, most patients had their device abandoned and received a new pacing system (72/85). The abandoned Micra was turned off in most patients and in a small minority was programmed as a back-up system. A new Micra was implanted in 13 patients and no interaction with the old Micra was seen and no evidence of interference with right ventricular function was noted. Ten patients had their system extracted successfully and a new system implanted with the oldest device extraction being a 4-year-old device. While these data suggest that devices with a mid-term dwell time can be extracted, removal of the device after complete encapsulation may be challenging due to the development of fibrotic tissue. Patients that receive Micra are typically older and have significant co-morbidities, therefore, avoiding an unnecessary and potentially risky extraction could be the preferred strategy at the time of upgrade or at the end of device life.

The 5-year mortality rate observed in the PAR (39.5%) is not unexpected. This cohort had an average age of 76 years with multiple co-morbidities including 21% of patients with chronic kidney disease, 8% on dialysis, and 26% with diabetes. It is possible that patients pre-selected for a leadless device are inherently a sicker patient population due to the perceived low-risk of infection with this technology. Furthermore, population-based studies have found greater co-morbidity to be associated with mortality, whereas survival among pacemaker recipients without significant co-morbidity approaches that of the general population.^19,20^

Only 12 patients had battery depletion occurring in the setting of elevated thresholds. The median projected battery longevity of patients by the end of 5 years of follow-up was 6.8 years and in line with the initial projection of a 12.1-year median battery life observed in the Micra VR IDE study.^5^ These findings are reassuring when it comes to the battery performance of Micra VR LP. The second-generation Micra VR has a projected battery longevity of 17 years possibly adding to the benefit of this technology.

### Limitations

While this study is derived from a prospective registry, it is not a randomized study and relies on comparison to a historical cohort that was constructed from six prior studies of dual-chamber pacing systems by excluding major complications and system revisions related to the right atrial lead. Patient baseline characteristics, including primary geographical location, were different between Micra VR patients and TV-PPM patients, however, after propensity matching, reductions in the risk for major complications and system revisions remained. Also, the historical TV-PPM cohort was only followed for 3 years as compared to 5 years with the LP group.

## Conclusions

This long-term follow-up of the Micra VR LP in > 1800 patients highlights the safety and reliability of this technology over a 5-year period, with low rates of complications and system revisions (both <5%) sustained through follow-up. In addition, complication and system revision rates were substantially lower than those of TV-PPM patients. The need for CRT upgrade was only 2% despite high burden of RV pacing. Notably, the incidence of infection was extremely low in this study. Long-term performance of the device will continue to be assessed in this ongoing trial.

## Supplementary data

Supplementary data are available at *European Heart Journal* online.

## Supplementary Material

ehae101_Supplementary_Data

## Declarations

### Data Availability

The data, analytical methods, and study materials are owned by the funder and will not be made available to other researchers for the purposes of reproducing the results or replicating the procedure.

### Pre-registered Clinical Trial Number

Registered at ClinicalTrials.gov, NCT number: NCT02536118.
