# Supplementary material for: Leadless pacemakers at 5-year follow-up: the Micra transcatheter pacing system post-approval registry
Source: Eur Heart J. 2024 Mar 1;45(14):1241–51. doi: 10.1093/eurheartj/ehae101 (PMC10998730; doi:10.1093/eurheartj/ehae101)
Supplement: ehae101_Supplementary_Data [file ehae101_supplementary_data.docx]

**Supplement**

***Supplementary Statistical Methods***

Propensity score overlap weights were used to account for differences in baseline co-morbidities between patients in the Micra VR PAR and patients in the historical TV-PPM cohort and construct adjusted hazard ratios for the risk for major complication and system revision through 36-months. For each patient, the propensity for receiving leadless pacing therapy was computed using a logistic regression model from the variables in Table S1. The resulting propensity scores were used to derive the overlap weight for each patient which could be used in weighted Fine-Gray models. Overlap weights range from 0 to 1 and place the most weight on patients that are most likely to receive either therapy. A feature of overlap weights is that when comparing two treatment groups the overlap weights induce exact balance on the means of the study population in the variables used to construct the propensity scores. Thus, while the weighting system utilizes data from all patients, some patients have lower weights than others in the adjusted analysis lowering the effective sample size.

Some patients in the historical TV-PPM cohort were missing co-morbidity status, particularly with respect to COPD, diabetes, and renal dysfunction. Rather than exclude these variables from our adjusted analysis, we used a fully conditional specification approach to impute values for the missing data. Specifically, we constructed 100 different imputed datasets and from each of these constructed propensity scores and overlap weights. Across our 100 different imputed datasets, the effective sample size for the transvenous control had a median of 2018 (range: 1979 – 2048) patients and the Micra VR group had an effective sample size with a median of 1524 (range: 1497 – 1543) patients.

For each of the 100 imputed datasets the absolute standardized mean differences (SMD) for each variable used to construct the propensity score were computed prior to weighting and after applying the overlap weights. **Supplementary Figure S4** shows that after applying the overlap weights the absolute SMDs were near zero for all variables included in the propensity score model indicating balance was achieved after weighting.

Following imputation, a single estimate for the hazard ratio and its 95% confidence interval were computed using Rubin’s rule. Propensity adjusted cumulative incidence plots for the major complication rate (**Supplementary Figure S5**) and system revision rate (**Supplementary Figure S6)** were computed by plotting the median value of the cumulative incidence function and its range across the 100 imputed datasets.

**Supplementary Table S1**: **Baseline characteristics and co-morbidity comparison between Micra VR PAR and historical transvenous pacemaker cohort**

| **Patient Characteristics** | **Micra PAR (N = 1809)** | **Transvenous Reference (N = 2667)** | **P-value** |
| --- | --- | --- | --- |
| **Age (years)** |  |  | **< 0.0001** |
| Mean ± Standard Deviation | 75.6 ± 13.4 | 71.1 ± 12.1 |  |
| Median | 79.0 | 73.5 |  |
| 25^th^ Percentile - 75^th^ Percentile | 71 - 84 | 65 - 80 |  |
| Minimum - Maximum | 13 - 101 | 9 - 100 |  |
| Number of Subjects with Measure Available (n, %) | 1807 (99.9%) | 2667 (100.0%) |  |
| **Female** | **38.8% (701/1808)** | **44.9% (1198/2667)** | **< 0.0001** |
| **Co-morbidities (%)** |  |  |  |
| AF | 71.2% (1287/1808) | 36.6% (977/2667) | < 0.0001 |
| CAD | 22.0% (398/1808) | 38.4% (1025/2667) | < 0.0001 |
| CHF | 13.1% (236/1808) | 15.0% (400/2667) | 0.074 |
| COPD | 9.8% (177/1808) | 7.2% (53/735) | 0.040 |
| Diabetes | 26.5% (479/1808) | 21.9% (395/1805) | 0.001 |
| Hypertension | 64.9% (1173/1808) | 67.2% (1792/2667) | 0.11 |
| Renal Dysfunction | 21.5% (389/1808) | 9.8% (26/266) | < 0.0001 |
|  | | | |

**Supplementary Table S2: Summary of death classification in Micra VR patients**

|  | **(n=1809)** | | |
| --- | --- | --- | --- |
| **Death Classification, No. Events (No. Patients, %)** | **Acute** | **Long-Term** | **Total** |
| **TOTAL DEATHS** | **30 (1.66%)** | **646 (35.71%)** | **676 (37.37%)** |
| **PROCEDURE/SYSTEM RELATED** | **5 (0.28%)** | **--** | **5 (0.28%)** |
| BLOOD PRESSURE DECREASED | 1 (0.06%) | -- | 1 (0.06%) |
| CARDIAC PERFORATION | 1 (0.06%) | -- | 1 (0.06%) |
| CARDIAC TAMPONADE | 1 (0.06%) | -- | 1 (0.06%) |
| PULMONARY OEDEMA | 1 (0.06%) | -- | 1 (0.06%) |
| RETROPERITONEAL HAEMORRHAGE | 1 (0.06%) | -- | 1 (0.06%) |
| **SUDDEN CARDIAC DEATH** | **2 (0.11%)** | **33 (1.82%)** | **35 (1.93%)** |
| ACUTE MYOCARDIAL INFARCTION | -- | 1 (0.06%) | 1 (0.06%) |
| ARTERIOSCLEROSIS | -- | 1 (0.06%) | 1 (0.06%) |
| ATRIAL FIBRILLATION | -- | 1 (0.06%) | 1 (0.06%) |
| CARDIAC ARREST | -- | 11 (0.61%) | 11 (0.61%) |
| CARDIAC DISORDER | -- | 1 (0.06%) | 1 (0.06%) |
| CARDIAC FAILURE | 1 (0.06%) | -- | 1 (0.06%) |
| CARDIAC FAILURE CHRONIC | -- | 1 (0.06%) | 1 (0.06%) |
| CARDIAC FAILURE CONGESTIVE | -- | 1 (0.06%) | 1 (0.06%) |
| CARDIO-RESPIRATORY ARREST | -- | 2 (0.11%) | 2 (0.11%) |
| CARDIOVASCULAR DISORDER | -- | 1 (0.06%) | 1 (0.06%) |
| DEATH | -- | 1 (0.06%) | 1 (0.06%) |
| DEVICE ELECTRICAL FINDING | -- | 1 (0.06%) | 1 (0.06%) |
| HYPOXIA | -- | 1 (0.06%) | 1 (0.06%) |
| LEFT VENTRICULAR FAILURE | -- | 1 (0.06%) | 1 (0.06%) |
| MULTIMORBIDITY | -- | 1 (0.06%) | 1 (0.06%) |
| MYOCARDIAL INFARCTION | -- | 3 (0.17%) | 3 (0.17%) |
| PULSELESS ELECTRICAL ACTIVITY | -- | 1 (0.06%) | 1 (0.06%) |
| SUDDEN CARDIAC DEATH | -- | 1 (0.06%) | 1 (0.06%) |
| VENTRICULAR ARRHYTHMIA | -- | 1 (0.06%) | 1 (0.06%) |
| VENTRICULAR FIBRILLATION | -- | 2 (0.11%) | 2 (0.11%) |
| VENTRICULAR TACHYCARDIA | 1 (0.06%) | -- | 1 (0.06%) |
| **NON-SUDDEN CARDIAC DEATH** | **2 (0.11%)** | **111 (6.14%)** | **113 (6.25%)** |
| ACUTE LEFT VENTRICULAR FAILURE | -- | 1 (0.06%) | 1 (0.06%) |
| ACUTE MYOCARDIAL INFARCTION | -- | 3 (0.17%) | 3 (0.17%) |
| ACUTE PULMONARY OEDEMA | -- | 1 (0.06%) | 1 (0.06%) |
| ACUTE RESPIRATORY DISTRESS SYNDROME | -- | 1 (0.06%) | 1 (0.06%) |
| ACUTE RESPIRATORY FAILURE | -- | 3 (0.17%) | 3 (0.17%) |
| AORTIC DISSECTION | -- | 1 (0.06%) | 1 (0.06%) |
| AORTIC VALVE STENOSIS | -- | 1 (0.06%) | 1 (0.06%) |
| ARTERIOSCLEROSIS CORONARY ARTERY | -- | 1 (0.06%) | 1 (0.06%) |
| CARDIAC ARREST | -- | 11 (0.61%) | 11 (0.61%) |
| CARDIAC DEATH | 1 (0.06%) | 3 (0.17%) | 4 (0.22%) |
| CARDIAC FAILURE | -- | 22 (1.22%) | 22 (1.22%) |
| CARDIAC FAILURE ACUTE | -- | 1 (0.06%) | 1 (0.06%) |
| CARDIAC FAILURE CHRONIC | -- | 9 (0.50%) | 9 (0.50%) |
| CARDIAC FAILURE CONGESTIVE | -- | 9 (0.50%) | 9 (0.50%) |
| CARDIO-RESPIRATORY ARREST | -- | 2 (0.11%) | 2 (0.11%) |
| CARDIOGENIC SHOCK | -- | 8 (0.44%) | 8 (0.44%) |
| CARDIOMYOPATHY | -- | 2 (0.11%) | 2 (0.11%) |
| CARDIOPULMONARY FAILURE | -- | 2 (0.11%) | 2 (0.11%) |
| CARDIORENAL SYNDROME | -- | 1 (0.06%) | 1 (0.06%) |
| CARDIOVASCULAR DISORDER | -- | 1 (0.06%) | 1 (0.06%) |
| DEATH | -- | 4 (0.22%) | 4 (0.22%) |
| END STAGE RENAL DISEASE | -- | 1 (0.06%) | 1 (0.06%) |
| ENDOCARDITIS | -- | 1 (0.06%) | 1 (0.06%) |
| HYPERTENSIVE HEART DISEASE | -- | 1 (0.06%) | 1 (0.06%) |
| ISCHAEMIC CARDIOMYOPATHY | -- | 1 (0.06%) | 1 (0.06%) |
| MULTIPLE ORGAN DYSFUNCTION SYNDROME | -- | 2 (0.11%) | 2 (0.11%) |
| MYOCARDIAL INFARCTION | -- | 1 (0.06%) | 1 (0.06%) |
| PARACENTESIS | -- | 1 (0.06%) | 1 (0.06%) |
| PNEUMONIA | -- | 1 (0.06%) | 1 (0.06%) |
| POST PROCEDURAL COMPLICATION | -- | 1 (0.06%) | 1 (0.06%) |
| PROSTHETIC VALVE ENDOCARDITIS | -- | 1 (0.06%) | 1 (0.06%) |
| PULMONARY EMBOLISM | -- | 1 (0.06%) | 1 (0.06%) |
| PULMONARY OEDEMA | -- | 1 (0.06%) | 1 (0.06%) |
| PULSELESS ELECTRICAL ACTIVITY | 1 (0.06%) | -- | 1 (0.06%) |
| RESPIRATORY FAILURE | -- | 3 (0.17%) | 3 (0.17%) |
| RIGHT VENTRICULAR FAILURE | -- | 1 (0.06%) | 1 (0.06%) |
| SEPSIS | -- | 1 (0.06%) | 1 (0.06%) |
| SEPTIC SHOCK | -- | 3 (0.17%) | 3 (0.17%) |
| UNEVALUABLE EVENT | -- | 1 (0.06%) | 1 (0.06%) |
| VENTRICULAR FIBRILLATION | -- | 2 (0.11%) | 2 (0.11%) |
| **NON-CARDIAC DEATH** | **14 (0.77%)** | **331 (18.30%)** | **345 (19.07%)** |
| ACUTE KIDNEY INJURY | -- | 5 (0.28%) | 5 (0.28%) |
| ACUTE MYELOID LEUKAEMIA | 1 (0.06%) | -- | 1 (0.06%) |
| ACUTE RESPIRATORY FAILURE | 1 (0.06%) | 6 (0.33%) | 7 (0.39%) |
| ADRENAL INSUFFICIENCY | 1 (0.06%) | -- | 1 (0.06%) |
| AORTIC ANEURYSM | -- | 1 (0.06%) | 1 (0.06%) |
| AORTIC ANEURYSM RUPTURE | -- | 1 (0.06%) | 1 (0.06%) |
| AORTIC DISSECTION | -- | 1 (0.06%) | 1 (0.06%) |
| ASPHYXIA | -- | 2 (0.11%) | 2 (0.11%) |
| ASPIRATION | -- | 1 (0.06%) | 1 (0.06%) |
| B-CELL LYMPHOMA | -- | 1 (0.06%) | 1 (0.06%) |
| BACTERAEMIA | -- | 4 (0.22%) | 4 (0.22%) |
| BLADDER CANCER | -- | 1 (0.06%) | 1 (0.06%) |
| BRAIN ABSCESS | -- | 1 (0.06%) | 1 (0.06%) |
| BRAIN STEM STROKE | -- | 1 (0.06%) | 1 (0.06%) |
| BREAST CANCER | -- | 2 (0.11%) | 2 (0.11%) |
| BREAST CANCER METASTATIC | -- | 1 (0.06%) | 1 (0.06%) |
| BREAST NEOPLASM | -- | 1 (0.06%) | 1 (0.06%) |
| BRONCHIAL ASPIRATION PROCEDURE | -- | 1 (0.06%) | 1 (0.06%) |
| CACHEXIA | -- | 1 (0.06%) | 1 (0.06%) |
| CARDIO-RESPIRATORY ARREST | -- | 2 (0.11%) | 2 (0.11%) |
| CEREBELLAR HAEMORRHAGE | -- | 1 (0.06%) | 1 (0.06%) |
| CEREBRAL ARTERIOSCLEROSIS | -- | 1 (0.06%) | 1 (0.06%) |
| CEREBRAL HAEMORRHAGE | -- | 2 (0.11%) | 2 (0.11%) |
| CEREBRAL INFARCTION | -- | 3 (0.17%) | 3 (0.17%) |
| CEREBROVASCULAR ACCIDENT | -- | 9 (0.50%) | 9 (0.50%) |
| CHOLANGIOCARCINOMA | -- | 1 (0.06%) | 1 (0.06%) |
| CHRONIC GASTROINTESTINAL BLEEDING | -- | 1 (0.06%) | 1 (0.06%) |
| CHRONIC KIDNEY DISEASE | -- | 2 (0.11%) | 2 (0.11%) |
| CHRONIC OBSTRUCTIVE PULMONARY DISEASE | -- | 4 (0.22%) | 4 (0.22%) |
| CHRONIC RESPIRATORY FAILURE | -- | 1 (0.06%) | 1 (0.06%) |
| CLOSTRIDIUM DIFFICILE COLITIS | -- | 1 (0.06%) | 1 (0.06%) |
| COLITIS | -- | 2 (0.11%) | 2 (0.11%) |
| COLON CANCER | -- | 1 (0.06%) | 1 (0.06%) |
| COMA HEPATIC | -- | 1 (0.06%) | 1 (0.06%) |
| COMPLETED SUICIDE | -- | 1 (0.06%) | 1 (0.06%) |
| CORONAVIRUS INFECTION | -- | 1 (0.06%) | 1 (0.06%) |
| COVID-19 | -- | 7 (0.39%) | 7 (0.39%) |
| COVID-19 PNEUMONIA | -- | 8 (0.44%) | 8 (0.44%) |
| DEATH | -- | 14 (0.77%) | 14 (0.77%) |
| DEHYDRATION | -- | 1 (0.06%) | 1 (0.06%) |
| DEMENTIA | -- | 1 (0.06%) | 1 (0.06%) |
| DEMENTIA ALZHEIMER'S TYPE | -- | 2 (0.11%) | 2 (0.11%) |
| DIFFUSE LARGE B-CELL LYMPHOMA | -- | 1 (0.06%) | 1 (0.06%) |
| DIVERTICULUM INTESTINAL HAEMORRHAGIC | -- | 1 (0.06%) | 1 (0.06%) |
| ELDERLY | -- | 2 (0.11%) | 2 (0.11%) |
| END STAGE RENAL DISEASE | -- | 6 (0.33%) | 6 (0.33%) |
| ENDOMETRIAL CANCER METASTATIC | -- | 1 (0.06%) | 1 (0.06%) |
| EUTHANASIA | -- | 1 (0.06%) | 1 (0.06%) |
| FAILURE TO THRIVE | -- | 1 (0.06%) | 1 (0.06%) |
| FALL | -- | 2 (0.11%) | 2 (0.11%) |
| FEMUR FRACTURE | -- | 1 (0.06%) | 1 (0.06%) |
| GANGRENE | -- | 1 (0.06%) | 1 (0.06%) |
| GASTRIC CANCER | -- | 1 (0.06%) | 1 (0.06%) |
| GASTRIC ULCER PERFORATION | -- | 1 (0.06%) | 1 (0.06%) |
| GASTROENTERITIS NOROVIRUS | -- | 1 (0.06%) | 1 (0.06%) |
| GASTROINTESTINAL HAEMORRHAGE | -- | 3 (0.17%) | 3 (0.17%) |
| GENERAL PHYSICAL HEALTH DETERIORATION | -- | 4 (0.22%) | 4 (0.22%) |
| HAEMORRHAGE INTRACRANIAL | -- | 3 (0.17%) | 3 (0.17%) |
| HAEMORRHAGIC FEVER WITH RENAL SYNDROME | -- | 1 (0.06%) | 1 (0.06%) |
| HAEMORRHAGIC STROKE | -- | 1 (0.06%) | 1 (0.06%) |
| HEPATIC CANCER | -- | 3 (0.17%) | 3 (0.17%) |
| HEPATIC CIRRHOSIS | -- | 2 (0.11%) | 2 (0.11%) |
| HEPATIC NEOPLASM | -- | 1 (0.06%) | 1 (0.06%) |
| HODGKIN'S DISEASE | -- | 1 (0.06%) | 1 (0.06%) |
| HYPERCAPNIA | -- | 1 (0.06%) | 1 (0.06%) |
| HYPERTENSION | -- | 1 (0.06%) | 1 (0.06%) |
| HYPOGLYCAEMIA | -- | 1 (0.06%) | 1 (0.06%) |
| HYPOPHARYNGEAL CANCER | -- | 1 (0.06%) | 1 (0.06%) |
| HYPOTHERMIA | -- | 1 (0.06%) | 1 (0.06%) |
| HYPOXIA | -- | 1 (0.06%) | 1 (0.06%) |
| INTERSTITIAL LUNG DISEASE | -- | 1 (0.06%) | 1 (0.06%) |
| INTESTINAL ISCHAEMIA | -- | 1 (0.06%) | 1 (0.06%) |
| INTESTINAL PERFORATION | -- | 1 (0.06%) | 1 (0.06%) |
| ISCHAEMIC STROKE | -- | 7 (0.39%) | 7 (0.39%) |
| LARYNGEAL NEOPLASM | -- | 1 (0.06%) | 1 (0.06%) |
| LARYNGEAL SQUAMOUS CELL CARCINOMA | -- | 1 (0.06%) | 1 (0.06%) |
| LEIOMYOSARCOMA | -- | 1 (0.06%) | 1 (0.06%) |
| LEUKAEMIA | -- | 1 (0.06%) | 1 (0.06%) |
| LUNG ADENOCARCINOMA | -- | 2 (0.11%) | 2 (0.11%) |
| LUNG CANCER METASTATIC | -- | 5 (0.28%) | 5 (0.28%) |
| LUNG CARCINOMA CELL TYPE UNSPECIFIED RECURRENT | -- | 1 (0.06%) | 1 (0.06%) |
| LUNG CARCINOMA CELL TYPE UNSPECIFIED STAGE IV | -- | 1 (0.06%) | 1 (0.06%) |
| LUNG DISORDER | -- | 3 (0.17%) | 3 (0.17%) |
| LUNG NEOPLASM MALIGNANT | -- | 7 (0.39%) | 7 (0.39%) |
| LYMPHADENOPATHY | -- | 1 (0.06%) | 1 (0.06%) |
| LYMPHOMA | -- | 3 (0.17%) | 3 (0.17%) |
| METASTASES TO BONE | -- | 1 (0.06%) | 1 (0.06%) |
| METASTASES TO LUNG | -- | 1 (0.06%) | 1 (0.06%) |
| METASTATIC MALIGNANT MELANOMA | -- | 1 (0.06%) | 1 (0.06%) |
| METASTATIC NEOPLASM | -- | 2 (0.11%) | 2 (0.11%) |
| MULTIPLE ORGAN DYSFUNCTION SYNDROME | -- | 8 (0.44%) | 8 (0.44%) |
| MYELODYSPLASTIC SYNDROME | -- | 3 (0.17%) | 3 (0.17%) |
| NECROTISING FASCIITIS | -- | 1 (0.06%) | 1 (0.06%) |
| NEOPLASM MALIGNANT | 1 (0.06%) | 1 (0.06%) | 2 (0.11%) |
| NEPHRITIS | -- | 1 (0.06%) | 1 (0.06%) |
| NEPHROPATHY | -- | 1 (0.06%) | 1 (0.06%) |
| NERVOUS SYSTEM DISORDER | -- | 2 (0.11%) | 2 (0.11%) |
| NEURODEGENERATIVE DISORDER | -- | 1 (0.06%) | 1 (0.06%) |
| OESOPHAGEAL CARCINOMA | -- | 1 (0.06%) | 1 (0.06%) |
| ORGAN FAILURE | -- | 1 (0.06%) | 1 (0.06%) |
| OVARIAN CANCER | -- | 1 (0.06%) | 1 (0.06%) |
| PANCREATIC CARCINOMA | -- | 2 (0.11%) | 2 (0.11%) |
| PANCREATITIS ACUTE | -- | 1 (0.06%) | 1 (0.06%) |
| PARKINSON'S DISEASE | -- | 1 (0.06%) | 1 (0.06%) |
| PELVIC HAEMATOMA | -- | 1 (0.06%) | 1 (0.06%) |
| PERIPHERAL ISCHAEMIA | -- | 1 (0.06%) | 1 (0.06%) |
| PERIPHERAL VASCULAR DISORDER | -- | 1 (0.06%) | 1 (0.06%) |
| PLASMA CELL MYELOMA | -- | 1 (0.06%) | 1 (0.06%) |
| PNEUMONIA | 2 (0.11%) | 25 (1.38%) | 27 (1.49%) |
| PNEUMONIA ASPIRATION | -- | 3 (0.17%) | 3 (0.17%) |
| PNEUMONIA PNEUMOCOCCAL | -- | 1 (0.06%) | 1 (0.06%) |
| PROSTATE CANCER | -- | 1 (0.06%) | 1 (0.06%) |
| PROSTATE CANCER METASTATIC | -- | 4 (0.22%) | 4 (0.22%) |
| PULMONARY SEPSIS | -- | 3 (0.17%) | 3 (0.17%) |
| REFRACTORY CYTOPENIA WITH UNILINEAGE DYSPLASIA | -- | 1 (0.06%) | 1 (0.06%) |
| REFUSAL OF TREATMENT BY PATIENT | -- | 1 (0.06%) | 1 (0.06%) |
| RENAL CANCER | -- | 1 (0.06%) | 1 (0.06%) |
| RENAL CANCER METASTATIC | -- | 1 (0.06%) | 1 (0.06%) |
| RENAL FAILURE | -- | 11 (0.61%) | 11 (0.61%) |
| RESPIRATORY ARREST | 1 (0.06%) | 2 (0.11%) | 3 (0.17%) |
| RESPIRATORY FAILURE | 3 (0.17%) | 9 (0.50%) | 12 (0.66%) |
| RESPIRATORY TRACT INFECTION | -- | 1 (0.06%) | 1 (0.06%) |
| RETROPERITONEAL HAEMATOMA | -- | 1 (0.06%) | 1 (0.06%) |
| ROAD TRAFFIC ACCIDENT | -- | 1 (0.06%) | 1 (0.06%) |
| SENILE DEMENTIA | -- | 1 (0.06%) | 1 (0.06%) |
| SEPSIS | 1 (0.06%) | 13 (0.72%) | 14 (0.77%) |
| SEPSIS SYNDROME | -- | 1 (0.06%) | 1 (0.06%) |
| SEPTIC SHOCK | 2 (0.11%) | 12 (0.66%) | 14 (0.77%) |
| SHOCK | 1 (0.06%) | 1 (0.06%) | 2 (0.11%) |
| SHOCK HAEMORRHAGIC | -- | 3 (0.17%) | 3 (0.17%) |
| SMALL CELL LUNG CANCER | -- | 1 (0.06%) | 1 (0.06%) |
| SMALL INTESTINAL OBSTRUCTION | -- | 1 (0.06%) | 1 (0.06%) |
| STATUS EPILEPTICUS | -- | 1 (0.06%) | 1 (0.06%) |
| SUBDURAL HAEMATOMA | -- | 3 (0.17%) | 3 (0.17%) |
| TRANSITIONAL CELL CANCER OF RENAL PELVIS AND URETE | -- | 1 (0.06%) | 1 (0.06%) |
| TYPE 1 DIABETES MELLITUS | -- | 1 (0.06%) | 1 (0.06%) |
| UNEVALUABLE EVENT | -- | 5 (0.28%) | 5 (0.28%) |
| UPPER GASTROINTESTINAL HAEMORRHAGE | -- | 2 (0.11%) | 2 (0.11%) |
| UPPER RESPIRATORY TRACT INFECTION | -- | 1 (0.06%) | 1 (0.06%) |
| URINARY TRACT INFECTION | -- | 1 (0.06%) | 1 (0.06%) |
| UROSEPSIS | -- | 3 (0.17%) | 3 (0.17%) |
| **UNKNOWN CLASSIFICATION** | **7 (0.39%)** | **171 (9.45%)** | **178 (9.84%)** |
| CARDIAC ARREST | -- | 1 (0.06%) | 1 (0.06%) |
| CARDIAC FAILURE | -- | 2 (0.11%) | 2 (0.11%) |
| CARDIAC FAILURE CONGESTIVE | 1 (0.06%) | -- | 1 (0.06%) |
| CARDIO-RESPIRATORY ARREST | 1 (0.06%) | 1 (0.06%) | 2 (0.11%) |
| COVID-19 PNEUMONIA | -- | 1 (0.06%) | 1 (0.06%) |
| DEATH | 4 (0.22%) | 131 (7.24%) | 135 (7.46%) |
| ELDERLY | -- | 1 (0.06%) | 1 (0.06%) |
| EUTHANASIA | -- | 1 (0.06%) | 1 (0.06%) |
| GENERAL PHYSICAL HEALTH DETERIORATION | -- | 1 (0.06%) | 1 (0.06%) |
| MULTIMORBIDITY | -- | 1 (0.06%) | 1 (0.06%) |
| PNEUMONIA | -- | 1 (0.06%) | 1 (0.06%) |
| RENAL CANCER | -- | 1 (0.06%) | 1 (0.06%) |
| SUDDEN DEATH | 1 (0.06%) | 2 (0.11%) | 3 (0.17%) |
| UNEVALUABLE EVENT | -- | 27 (1.49%) | 27 (1.49%) |
|  | | | |

**Supplementary Table S3**: **Summary of system revisions in the historical TV-PPM cohort**

|  | **Revisions (Patients)** |
| --- | --- |
| **Total System Revisions** | **138 (128)** |
| **System Component Modified** |  |
| Device and RV lead | 31 (30) |
| Device only | 17 (16) |
| RV lead only | 90 (86) |
| **Reason for System Revision** |  |
| Cardiac perforation | 7 (7) |
| Device migration | 2 (2) |
| Device upgrade | 15 (15) |
| High threshold | 28 (26) |
| Infection | 8 (8) |
| Lead dislodgement | 41 (41) |
| Lead failure | 3 (3) |
| Pacemaker syndrome | 1 (1) |
| Phrenic nerve stimulation | 7 (7) |
| Pneumothorax | 1 (1) |
| Pocket site pain | 3 (3) |
| Other | 8 (8) |
| Not reported | 14 (13) |
|  | |

**Supplementary Figure S1: All-cause mortality through 5-years.** All-cause mortality rate computed through 5-years using the Kaplan-Meier method.

**
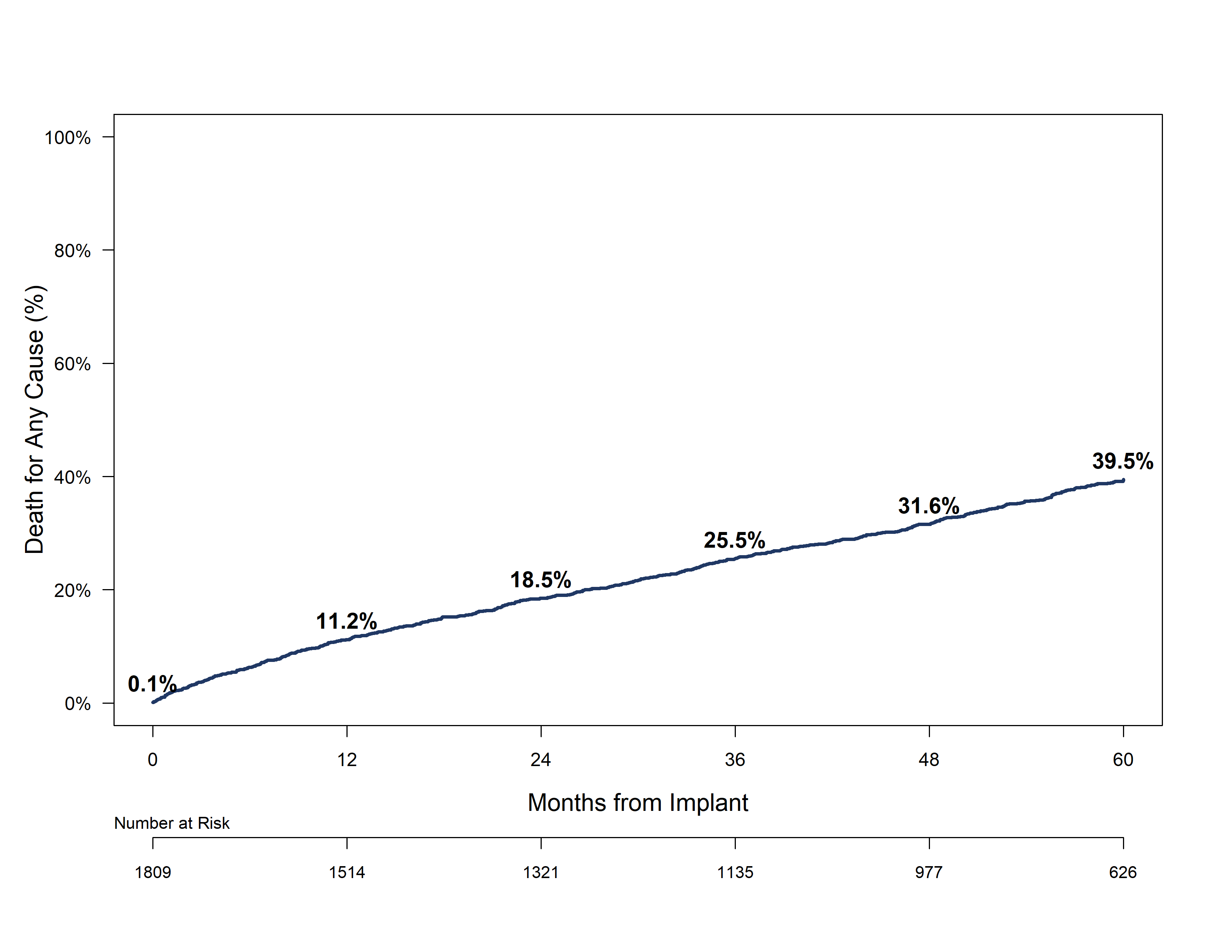
**

**Supplementary Figure S2: CRT Upgrade Rate through 5-years.** CRT upgrade rate through 5-years estimated using the cumulative incidence function under the competing risk of death.

**
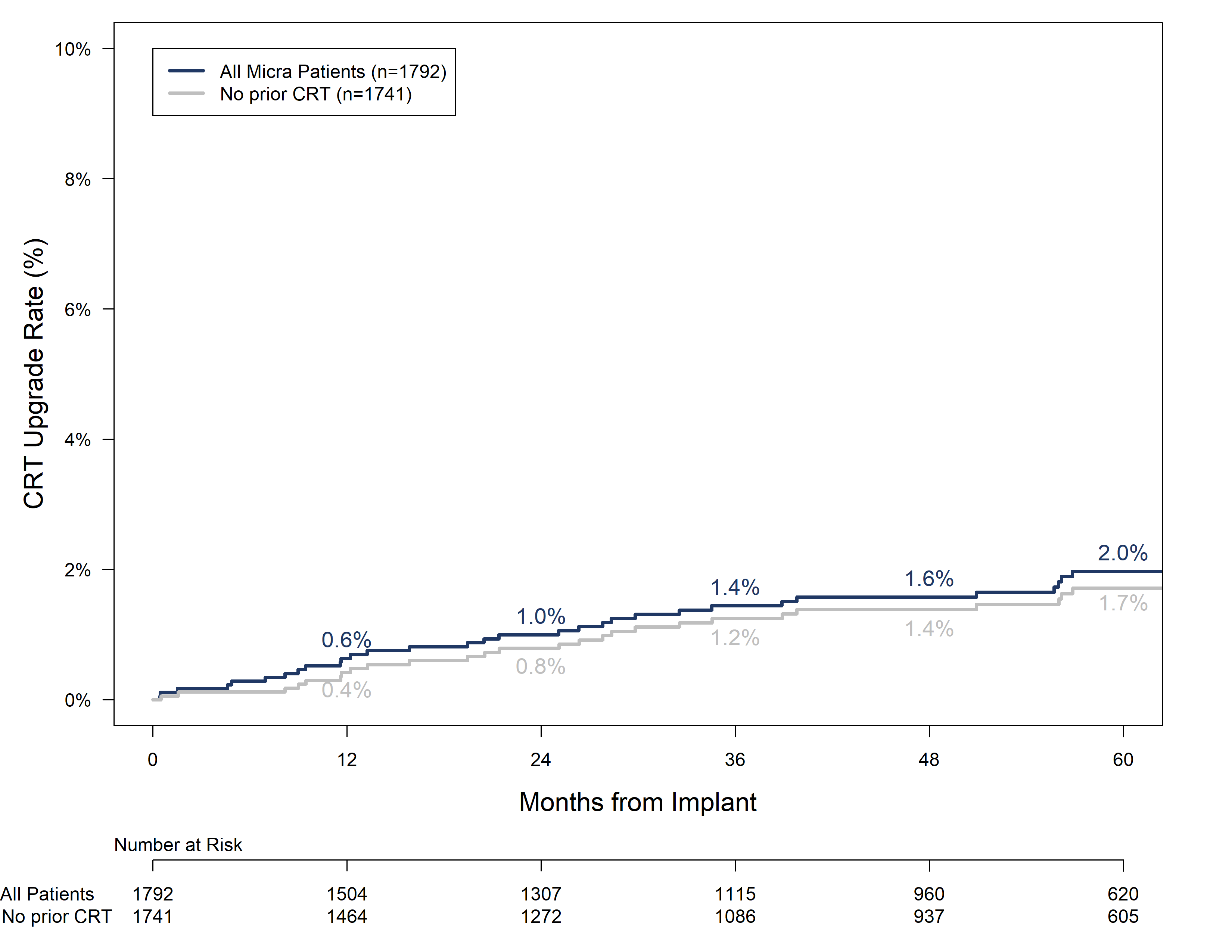
**

**Supplementary Figure S3: Cumulative Rate of Micra VR Devices Out of Service by Reason through 60-months.** Death means patient had an active Micra device at the time of patient death. Elevated pacing capture threshold (PCT)/Battery Depletion means that the device was taken out of service for elevated PCT or for reaching its elective replacement interval. Other out of service reason means the device was taken out of service for other reasons such as need for device upgrade.

**
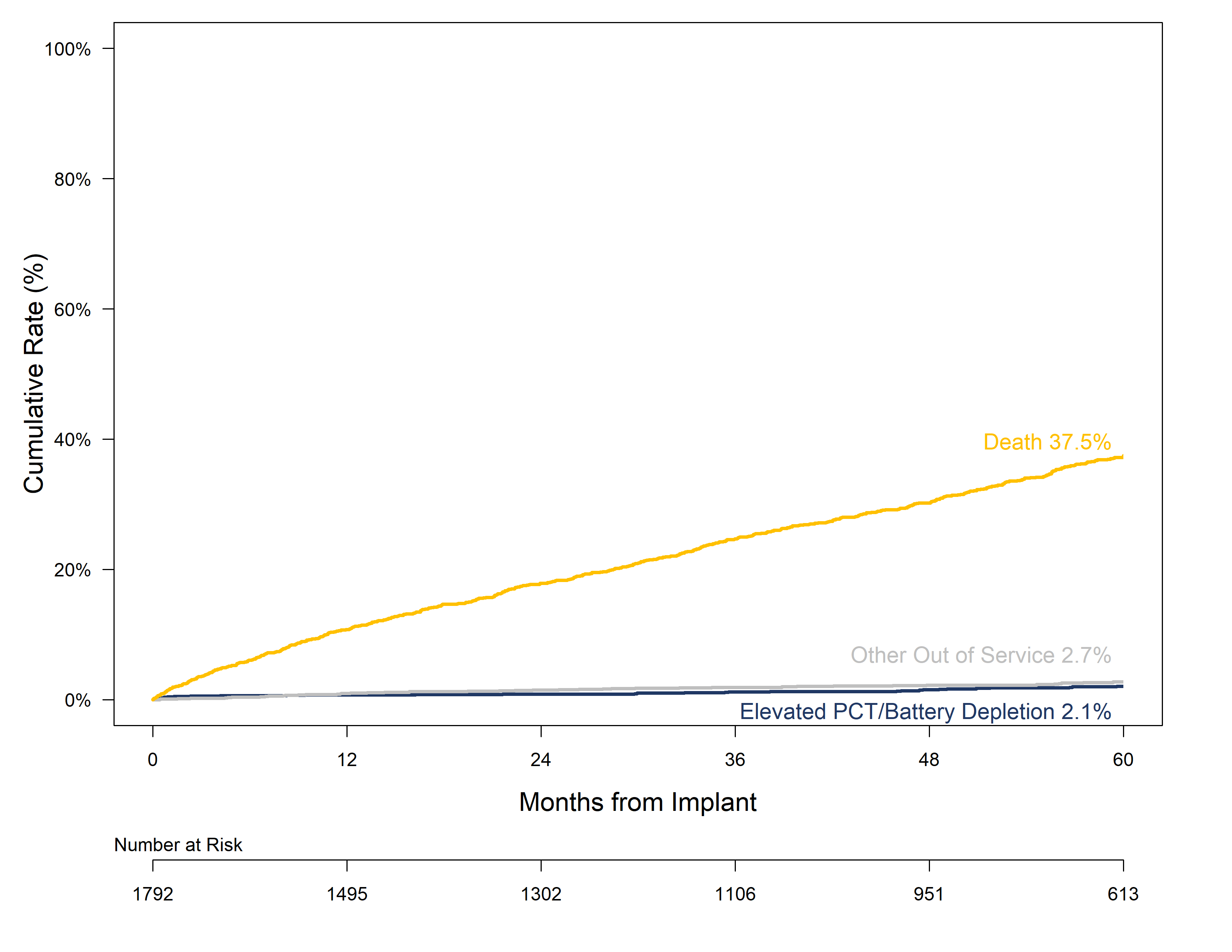
**

**Supplementary Figure S4: Absolute Standardized Mean Difference in Baseline and Co-morbidity Variables used to Construct Propensity Score Overlap Weights**

**
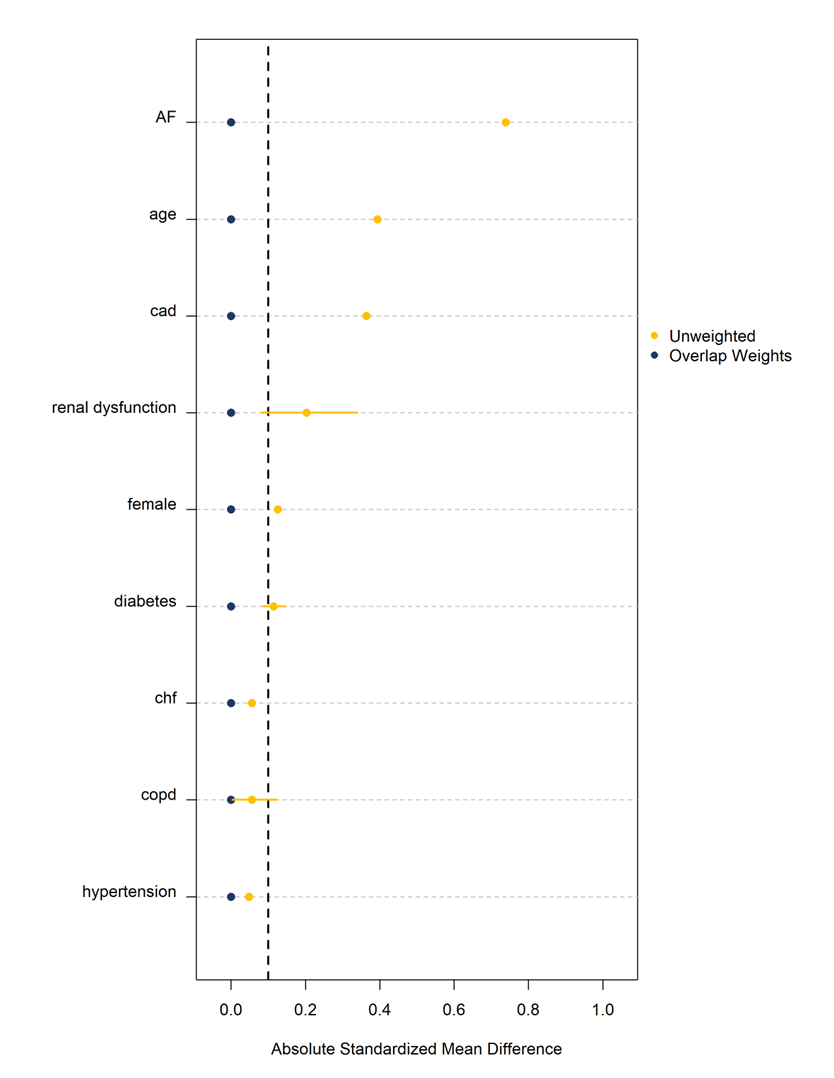
**

Note: error bars in the plot indicate the range (min to max) of the standardized mean difference across the 100 imputed datasets. The highest variability in the standardized mean differences for the unweighted data were associated with the variables with the greatest amount of missing data.

**Supplementary Figure S5:** **Propensity Adjusted Rate of Major Complications Since Implant**

**
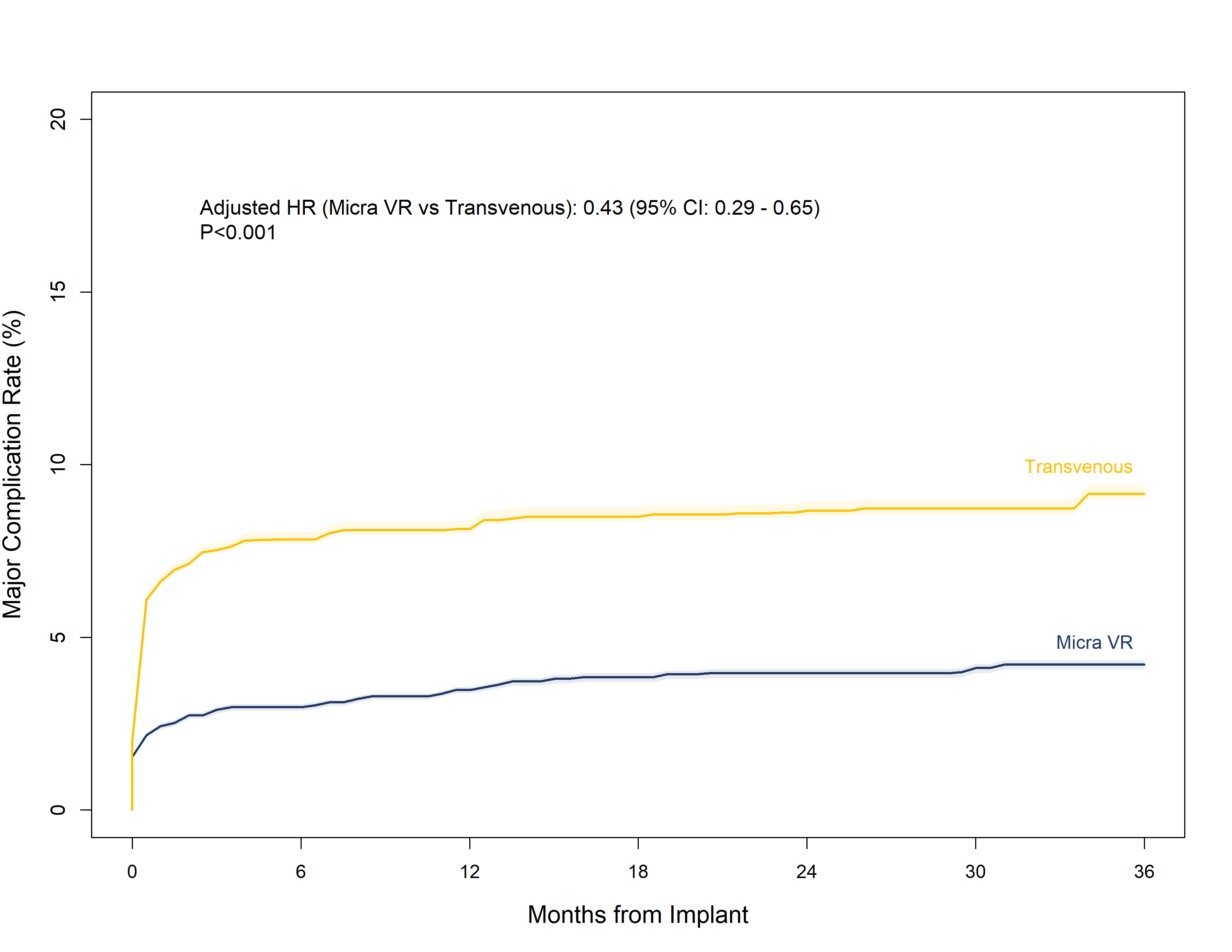
**

Notes: Adjusted hazard ratio from Fine-Gray model using overlap weights to adjust for differences in baseline characteristics. Solid line connects median adjusted rate at each timepoint across the 100 imputed datasets. Shaded region covers the minimum and maximum rates across the 100 imputed datasets.

**Supplementary Figure S6:** **Propensity Adjusted Rate of System Revision for Any Reason Since Implant**

**
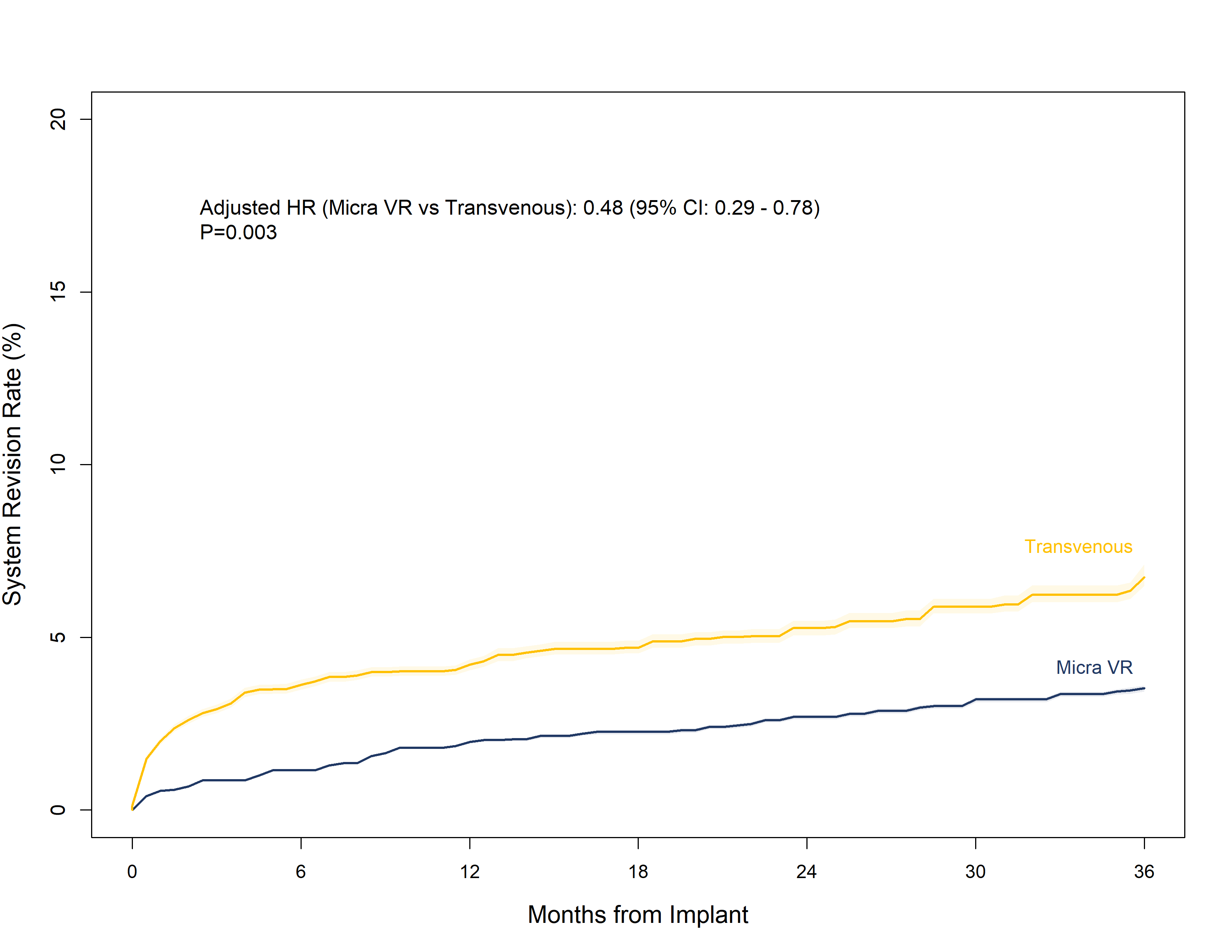
**

Notes: Adjusted hazard ratio from Fine-Gray model using overlap weights to adjust for differences in baseline characteristics. Solid line connects median adjusted rate at each timepoint across the 100 imputed datasets. Shaded region covers the minimum and maximum rates across the 100 imputed datasets.
